# Early Childhood Anemia in Ghana: Prevalence and Predictors Using Machine Learning Techniques

**DOI:** 10.3390/children12070924

**Published:** 2025-07-12

**Authors:** Maryam Siddiqa, Gulzar Shah, Mahnoor Shahid Butt, Asifa Kamal, Samuel T. Opoku

**Affiliations:** 1 Department of Mathematics & Statistics, International Islamic University Islamabad, Islamabad 44000, Pakistan; maryam.siddiqa@iiu.edu.pk (M.S.); mahnoor.msst202@student.iiu.edu.pk (M.S.B.); 2 Department of Health Policy and Management, Jiann-Ping Hsu College of Public Health, Georgia Southern University, Statesboro, GA 30460, USA; gshah@georgiasouthern.edu; 3 Department of Statistics, Lahore College for Women University, Lahore 54000, Pakistan; asifa.kamal@lcwu.edu.pk

**Keywords:** anemia, children, machine learning

## Abstract

**Background/Objectives**: Early childhood anemia is a severe public health concern and the most common blood disorder worldwide, especially in emerging countries. This study examines the sources of childhood anemia in Ghana through various societal, parental, and child characteristics. **Methods**: This research used data from the 2022 Ghana Demographic and Health Survey (GDHS-2022), which comprised 9353 children. Using STATA 13 and R 4.4.2 software, we analyzed maternal, social, and child factors using a model-building procedure, logistic regression analysis, and machine learning (ML) algorithms. The analyses comprised machine learning methods including decision trees, K-nearest neighbor (KNN), logistic regression, and random forest (RF). We used discrimination and calibration parameters to evaluate the performance of each machine learning algorithm. **Results**: Key predictors of childhood anemia are the father’s education, socioeconomic status, iron intake during pregnancy, the mother’s education, and the baby’s postnatal checkup within two months. With accuracy (94.74%), sensitivity (82.5%), specificity (50.78%), and AUC (86.62%), the random forest model was proven to be the most effective machine learning predictive model. The logistic regression model appeared second with accuracy (67.35%), sensitivity (76.16%), specificity (56.05%), and AUC (72.47%). **Conclusions**: Machine learning can accurately predict childhood anemia based on child and paternal characteristics. Focused interventions to enhance maternal health, parental education, and family economic status could reduce the prevalence of early childhood anemia and improve long-term pediatric health in Ghana. Early intervention and identifying high-risk youngsters may be made easier with the application of machine learning techniques, which will eventually lead to a healthier generation in the future.

## 1. Introduction

Anemia is a significant global public health issue, particularly in emerging countries [1,2]. It primarily affects children under five and pregnant women due to micronutrient deficiencies of vitamin B12 and folate, as well as an inadequate intake of iron-rich foods [3,4]. About 39.8% of children 5 years or below and 36.5% of pregnant women are anemic worldwide [3,5]. In Africa, the prevalence of anemia is 67.6% among children aged 5 years or below [6]. According to WHO estimates, prevalence among children under five might reach 68% [7]. Ghana, a country in West Africa, has an anemia rate of 54.5% among children in early childhood, with notable differences between rural and urban areas, 59.1% vs. 41.1%, respectively [8]. According to WHO guidelines, anemia is considered a public health problem when the prevalence reaches 5%. The public health significance is mild between 5% and 19%, moderate between 20% and 29%, and severe at 40% [8]. The most prevalent type of anemia is iron deficiency anemia (IDA) [9]. IDA is expected to be the basis of almost half of all anemia instances and the cause of one million deaths per year worldwide [9,10,11]. Nevertheless, there remains a lack of accurately documented and biochemically evaluated IDA prevalence based on representative population-based samples in the African region.

Anemia occurs when there is a shortage of red blood cells, a reduction in their size, or a decrease in the hemoglobin concentration below normal. This illness may make it more difficult for the blood to carry oxygen throughout the body efficiently [12]. The most pronounced group of the affected population, due to the absence of red blood cells, is pregnant women and children under 5 years of age, who need more iron for better growth [13]. The primary reason for anemia in children under five years old is insufficient iron [14,15], vitamin B12 (cyanocobalamin), and vitamin B9 (folate), increasing the risk of death [16,17]. A low consumption of healthy food, the presence of worms, the mother’s history of anemia, problems with the intestines, and a lack of awareness are additional risk factors [18]. Anemia and malnutrition have serious long-term consequences on children’s growth, development, and overall health [19,20].

In many African nations, anemia is mainly caused by parasite infection, which increases the breakdown of red blood cells [21,22]. According to recent research, over 49% of Ghanaian children between the ages of 6 and 59 months suffer from anemia, with mild anemia accounting for 27.6% and moderate to severe anemia accounting for 21.4%. This is consistent with results from the Ghana Demographic and Health Survey of 2022, which also showed a 49% prevalence in this age range [23]. While child anemia remains an uncontrolled health concern in undeveloped and underprivileged nations, it has a significant public health impact and high prevalence in low-resourced countries [24,25].

In addition to dietary deficiencies, childhood anemia arises from the interaction of several factors at different influencing levels, including maternal characteristics, child traits, and individual biological and social factors [26,27]. Children’s anemia rates are higher in areas where infectious illnesses like malaria and hookworm infections are common because they seriously affect iron intake and metabolism [26,27,28]. Chronic anemia results from genetic diseases impacting hemoglobin production and function [29]. Social determinants also affect anemia. For instance, anemia is more common among children from lower socioeconomic families, those with low levels of mother’s education, household income, and availability of medical treatment [27,30,31]. Other risk factors for childhood anemia include maternal age, nutritional status, and underlying medical problems (such as maternal anemia) [26,28,32]. Premature or underweight babies are more likely to suffer from anemia because their physiological systems are not fully mature, which can impact how well they absorb and use nutrients [30].

Applications for machine learning in the medical domain are growing. The two fields that benefit from the use of ML are illness diagnosis and outcome prediction [33,34]. While conventional statistical approaches try to identify relationships between variables, machine learning algorithms concentrate on generating accurate and practical predictions [35]. Unlike traditional statistical approaches, machine learning approaches are data-driven and free from strict assumptions [36]. In the healthcare industry, machine learning (ML) techniques outperform conventional techniques in health prediction. The choice between machine learning (ML) techniques and logistic regression depends on the specific problem, data, and goals. ML models often achieve higher accuracy than logistic regression, especially with large datasets.

There is a lack of comprehensive and updated research evidence concerning child-related, parental, and societal predictors of early childhood anemia in Ghana. Therefore, it is imperative to assess inconsistencies in the children of Ghana to identify the determinants and barriers to improving child anemia rates. This study contributes to the literature on this topic by examining the predictors of child anemia in Ghanaian children using conventional logistic regression and machine learning algorithms. We pursue three research questions: (1) Which social determinants of health operationalized through household sociodemographic characteristics are associated with childhood anemia? (2) Which maternal characteristics are associated with childhood anemia? (3) Which child characteristics predict childhood anemia? The study’s findings yield critical research evidence for evidence-based public health practice and policy.

## 2. Materials and Methods

### 2.1. Dataset and Study Population

The Ghana Demographic and Health Survey (GDHS), a nationally representative household survey, provided the data for this study. Data on various demographic topics, including women’s and children’s health and nutritional status, are collected through the survey, which is conducted every five years. The funding for the GDHS was provided by the Government of Ghana, the United States Agency for International Development (USAID), the U.S. President’s Malaria Initiative (PMI), the United Nations Population Fund, UNFPA, UNICEF, the World Bank, the Global Fund, the Korean International Cooperation Agency (KOICA), and the World Health Development Office (UK-FCDO) and World Health Organization (WHO). The ICF provided technical assistance through the DHS Program, a USAID-funded initiative that supports the implementation of demographic and health surveys in countries worldwide. The GDHA used a two-stage stratified cluster sampling approach. In the initial step, clusters were chosen as the primary sampling units. Households, the secondary sampling units, were selected in the second phase. From the households in the sample, children aged 6 to 60 months had their hemoglobin levels tested. Interviews were conducted with 7044 males aged 15–59 in half of the chosen houses and 15,014 women aged 15–49 in 17,933 households, representing a nationally representative sample. Estimates for the 16 regions of the nation, as well as for urban and rural areas, are provided by the sample design for the 2022 Ghana Demographic and Health Survey (GDHS). To address the missing data, we employ the multiple imputation approach. The analysis is predicated on established cases. For descriptive results, a weighted sample of 9353 children aged 6 to 60 months was analyzed in this research.

### 2.2. Study Variables and Measurements

Childhood anemia status is the outcome variable. As part of the GDHS survey, blood samples were collected from children aged 6 to 60 months for anemia testing. Consent was obtained from their parents or other responsible adults. A microcuvette was used to collect blood samples. A portable battery-operated HemoCue analyzer (HemoCue, Ängelholm, Sweden) was used for hemoglobin analysis, and the findings were provided on the spot. Anemia was quantified as a binary outcome variable for this research. As a result, children with anemia were assigned a value of 1, and those without it were assigned a value of 0. If a child’s altitude-adjusted hemoglobin level was less than 11.0 g/dL, they were considered anemic [37].

The twenty-seven predictor variables (features) used in this study were selected from prior research conducted in Ghana and other locations based on their association with childhood anemia. These are region (Wester, Central, Greater Accra, Volta, Eastern, Ashanti, Western North, Ahafo, Bono, Bono East, Oti, Northern, Savannah, North East, Upper East, or Upper West), the place of residence (urban or rural), household members (<4, 4–6, 7–9, or >9), the source of drinking water (unimproved or improved), the sex of the household head (female or male), the father's education (no education, primary, secondary, or higher), socioeconomic status (poor, middle, or rich), the mother's education (no education, primary, secondary, or higher), maternal age (15–19, 20–24, 25–29, 30–34, 35–39, 40–44, or 45–49), maternal smoking (no or yes), whether the child was ever breastfed (no or yes), the initiation of breastfeeding (immediately, within first hour, or within first day), the mother's occupation (not working or working), the intake of iron during pregnancy (no or yes), the consumption of drugs for intestinal parasites during pregnancy (no or yes), birth order number (first born, 2–4, or >5), birth type (single or multiple birth), the sex of the child (male or female), the size of the child at birth (small, average, or large), formula milk consumption (no or yes), the child's age in months (0–6, 7–12, 13–24, 25–36, 37–48, or 49–60), stunting (no, moderate, or severe), wasting (no, moderate, or severe), underweight (no, moderate, or severe), the intake of fruits and vegetables (no or yes), baby postnatal checkup within 2 months (no or yes), and whether the child was given zinc (no or yes).

### 2.3. Statistical Analysis

SPSS version 20 and STATA version 13 were used for data cleaning and descriptive analyses, and R 4.4.1 was used to apply machine learning techniques to assess childhood anemia. Four machine learning methods, including decision trees (DTs), random forest (RF), K-nearest neighbor (KNN), and logistic regression (LR), were employed to identify the most significant predictors of early childhood anemia in Ghana. The performance of these different machine learning algorithms was then evaluated. Our dataset is relatively small, categorized as a moderate to large dataset. Datasets of such sizes can perform well with our chosen techniques. Secondly, our chosen algorithms strike a balance between model complexity and interpretability, which is vital for understanding the relationships between the predictors and the outcome variable. Our chosen algorithms are computationally efficient and can be run on standard hardware, ensuring essential reproducibility.

The Machine Learning (ML) Specific Checklist has been followed, and this study is well-planned, executed, and evaluated. We have clearly defined the problem and determined the goal by specifying the questions we are trying to answer. At the first stage of data preparation, the dataset was checked for errors, inconsistencies, and missing values. The data were preprocessed for feature scaling and coding after splitting them into training and testing sets. At the model selection stage, we identified the problem type as a classification and selected suitable models, including random forest, logistic regression, decision trees, and K-nearest neighbor. Model evaluation was based on appropriate metrics, such as the ROC curve, accuracy, sensitivity, specificity, positive predictive value, negative predictive value, F1 score, SHAP plot, and partial dependence plot. Following this, discrimination and calibration techniques were employed to assess the model’s performance.

Discrimination and calibration techniques were used to assess the models’ prediction ability [38]. The degree of agreement between each model’s predicted probabilities of anemia’s presence and observed anemia frequencies was measured using calibration plots; the models’ discrimination was evaluated using the area under the curve, which was estimated using the receiver characteristic curve; each model’s accuracy, sensitivity, specificity, positive predictive value, and negative predictive value were obtained and determined the most significant predictors of childhood anemia based on the most accurate algorithms.

## 3. Results

Table 1 summarizes descriptive and univariate statistics on risk factors associated with childhood anemia. The table is divided into three categories: societal characteristics, parental characteristics, and child characteristics. The odds ratios (OR) and 95% confidence intervals (CI) for several traits associated with childhood anemia are also presented in this table. Around 93% of households have more than nine members. Approximately 59% of households reside in rural areas. A substantial proportion of households, 41%, obtained their drinking water from sources that were not improved. About three-fourths (73%) of households were led by males. Approximately 65% of fathers had some formal education. About 56% of the children have poor socioeconomic status. Approximately 16% of mothers have only completed primary education. Approximately 24% of the mothers belonged to the 20–29 age group. Women’s smoking ratio was reported to be less than 1% in Ghana. About half (54%) of the pregnant women consumed intestinal parasite medication, and most (91%) mothers consumed iron during pregnancy. More than half (55.15%) of children were breastfed, and 64% were nursed within the first hour of birth. In Ghana, most mothers (83%) are employed.

The explanatory factors that are substantially connected with anemia status in the country of Ghana include region, the place of residence, the sex of the household head, the father’s education, socioeconomic level, the mother’s education, maternal age, whether the child was ever breastfed, the mother's occupation, the intake of iron during pregnancy, taking medicines to treat intestinal parasites when pregnant, birth order number, the sex of the child, the child’s age in months, the intake of fruits and vegetables, stunting, and infant postnatal check within 2 months; these had a *p* value less than 0.05. Children in the Northern region are more likely to be anemic (OR = 1.996; 95% CI, 1.646 to 2.345). Children residing in rural areas had a higher risk of being anemic (OR = 1.516; 95% CI, 1.335 to 1.722) compared to children from urban families. The risks of childhood anemia are substantially reduced in households headed by women (OR = 0.831; 95% CI, 0.722 to 0.956). The children of fathers with higher formal education have lower chances of developing anemia (OR = 0.485; 95% CI, 0.390 to 0.603) compared to the children whose fathers are uneducated.

Children from a higher socioeconomic status had a lower chance of being anemic (OR = 0.449; 95% CI, 0.386 to 0.522) compared to those from a lower socioeconomic status. A child with a highly educated mother was less likely to be anemic (OR = 0.400; 95% CI, 0.309 to 0.519) than a child with an uneducated mother. Compared to mothers who are between the ages of 15 and 19, children whose mothers are between the ages of 35 and 39 are less likely to become anemic (OR = 0.541; 95% CI, 0.362 to 0.809). Children of working mothers were less likely to develop anemia (OR = 0.756; 95% CI, 0.632 to 0.904) compared to those of non-working mothers. Mothers who take iron during pregnancy have children who have a lower chance of developing anemia (OR = 0.575; 95% CI, 0.410 to 0.807) as compared to mothers who do not take iron during pregnancy. The prevalence of anemia is lower in children whose mothers consume drugs for intestinal parasites (OR = 0.680; 95% CI, 0.568 to 0.814). A child with a birth order of five or higher has a greater chance of developing anemia (OR = 1.476; 95% CI, 0.999 to 1.355) as compared to the first child. Female children have a lower chance of developing anemia (OR = 0.854; 95% CI, 0.753 to 0.968). Children aged 49 to 60 months are less likely to be anemic (OR = 0.342; 95% CI, 0.217 to 0.539) than those aged 0 to 6 months. A child with moderate stunting is less likely to develop anemia than a child suffering from severe stunting (OR = 1.423; 95% CI, 0.301–1.594). A child who consumes fruits and vegetables is less likely to become anemic (OR = 0.268136; 95% CI, 0.055868 to 1.523078) than those who do not eat fruits and vegetables. A child having a postnatal checkup within two months is less likely to develop anemia (OR = 0.5728842; 95% CI, 0.3585152 to 0.9154322).

Table 2 shows the multivariate analysis of anemia status for children 5 years or below in Ghana. The father’s education, socioeconomic status, mother’s education, baby’s postnatal checkup within 2 months, and iron intake during pregnancy proved significant characteristics in Ghana (*p* < 0.05). The results show that a father’s education is the first important variable associated with childhood anemia in Ghana. Compared to fathers with no formal education, the odds of anemia are lower for kids if the father had secondary education (AOR = 0.652; 95% CI: 0.288 to 2.126) or a higher level of education (AOR = 0.156; 95% CI: 0.182 to 5.457). The study established that middle-class households were less likely to have anemic children (AOR = 0.010; 95% CI: 0.0001 to 0.568) than low-income households. The children of low-income families are more likely to be anemic than those of middle-income families. Additionally, the children of highly educated mothers (AOR = 0.002; 95% CI: 0.0009 to 0.529) are less likely to be anemic than those of mothers with only an elementary or secondary education. One of the most critical factors that might affect children’s anemic status is their initial period of breastfeeding. Children fed immediately after birth are less likely to develop anemia (AOR = 3.445; 95% CI: 1.540 to 7.313) than children fed within the first hour. Additionally, iron consumption during pregnancy is a significant contributing factor to childhood anemia. Compared to mothers who do not take iron throughout pregnancy, those who do have a lower risk of developing anemia (AOR = 0.017; 95% CI: 0.004 to 6.122). Childhood anemia is associated with a baby’s postnatal examination within two months. Children who receive a postnatal checkup within two months are less likely to be anemic (AOR = 0.732; 95% CI: 3.452 to 8.076) than children who do not receive a postnatal exam within two months.

**Figure 1:**
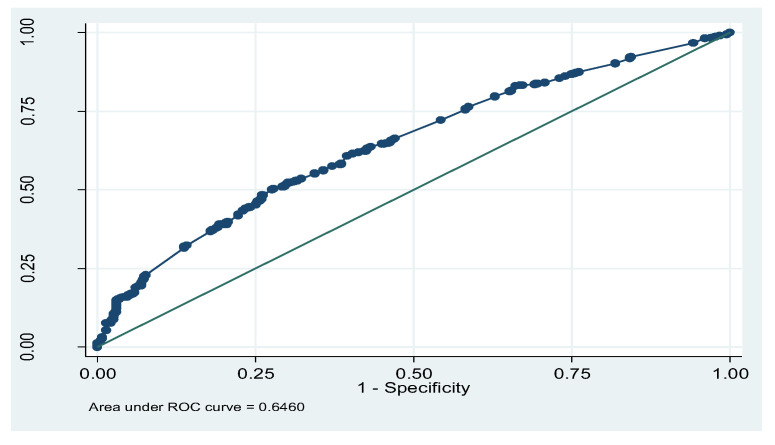
Receiver operating curve (ROC) of the logistic regression model.

Figure 1 illustrates the predictive quality of logistic regression models. The total area under the receiver operating curve (ROC) is 64.60% for the logistic regression model. Moreover, the ROC curve is closer to the upper left diagonal, which specifies that the model performed well.

Table 3 displays the performance of the machine learning algorithms built on training data. After training the dataset, the four machine learning algorithms projected childhood anemia, as shown in Table 4. We used a confusion matrix to evaluate the prediction performance of the algorithms. According to the confusion matrix, the random forest model accurately predicted that 1387 children would have anemia and 280 would not. It was incorrectly expected that 772 children would not have anemia, and 366 would. With an AUC of 98% (95% CI: 99.55–99.93), a sensitivity of 98% (95% CI: 99.35–99.93), a specificity of 67% (95% CI: 99.29–99.95), a classification accuracy of 95% (95% CI: 99.48–99.89), and an F1 Score of 98.20%, the random forest model exceeds the other models in predicting childhood anemia. The second-best algorithm for predicting anemia in children was the logistic regression algorithm. The K-nearest neighbor algorithm provided the least accuracy among all the algorithms used. The random forest model performed better than the other models. Given its capacity to manage complex feature interactions and minimize overfitting, random forest appears to be a good fit for this challenge.

**Figure 2:**
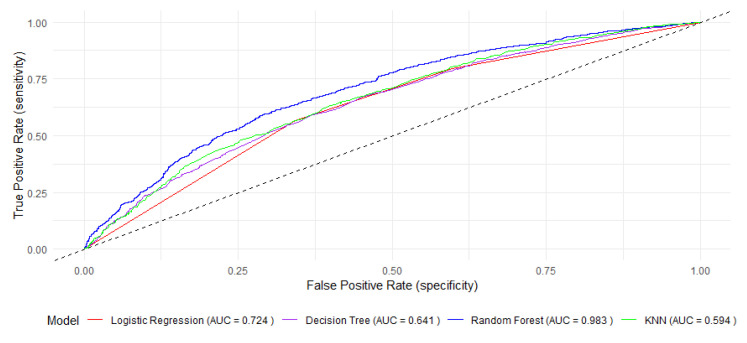
Receiver operating curves (ROCs) for each of the four ML models.

Additionally, the receiver operating characteristics curves (ROCs) for each algorithm are shown in Figure 2. The area under the curve (AUC) is a measure of the overall performance of the classifier. The random forest model presents the largest AUC value (98%) among the four machine learning models utilized in this investigation. It suggests that it is the most effective in discriminating between children with and without anemia.

**Figure 3:**
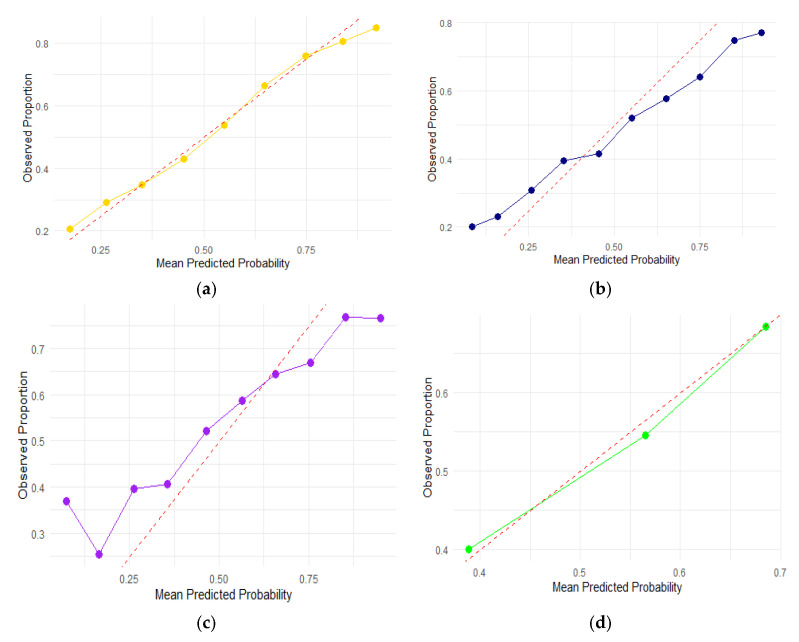
Calibration plots for each of the four ML models. (**a**) Calibration plot for random forest; (**b**) calibration plot for logistic regression; (**c**) calibration plot for KNN; and (**d**) calibration plot for decision tree.

To illustrate the accuracy of the four models, we created calibration plots for each of the four ML models shown in Figure 3. The calibration figure shows the degree of agreement between the observed anemia frequency (*Y*-axis) and the average predicted probability of anemia predicted by the models (*X*-axis). The graphical representation in Figure 3a demonstrates that the calibration by the random forest algorithm is good, as the mean predicted probability of childhood anemia is comparable to the actual childhood anemia rate across the entire dataset distribution. The logistic regression algorithm appeared to be the second-best fit. The measured frequency differs significantly from the predictions made by the other models. A *p* value of 0.98 for the Hosmer–Lemeshow test and 0.72 for the Pearson chi-square goodness-of-fit test indicated that the random forest model accurately describes the data.

**Figure 4:**
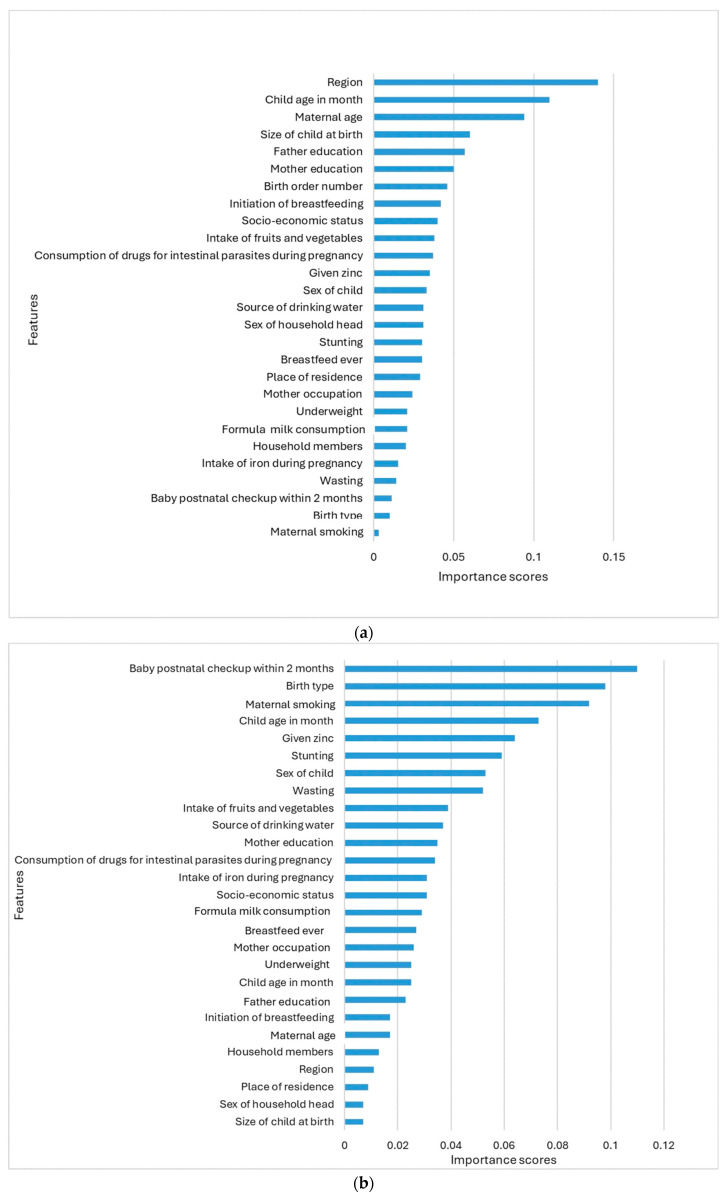
(**a**) Important features from the random forest algorithm. (**b**) Important features from the logistic regression algorithm.

Figure 4a shows the essential features of the random forest model. The features are ranked based on their importance scores, with the most critical features at the top. The vital score measures the contribution of each feature to the model’s prediction accuracy. The features of importance scores are displayed as probability values, which range from zero to one. The region has the highest importance score, indicating that it makes the most significant contribution to the model’s prediction accuracy. The child’s age in months has the second-highest importance score, suggesting that it plays a substantial role in model prediction. Maternal age is also quite important, ranking third in the model. The size of the child at birth is slightly less critical, but it still makes a significant contribution to the model. Maternal smoking has the lowest importance score, suggesting it has the least impact on the model outcomes.

Figure 4b displays the variable importance scores for the logistic regression algorithm, which include postnatal checkup, birth type, maternal smoking, the child's age, whether the child was given zinc, stunting, and the child’s sex. In contrast, the sex of the household head, birth size, place of residence, and region are the least important variables.

**Figure 5:**
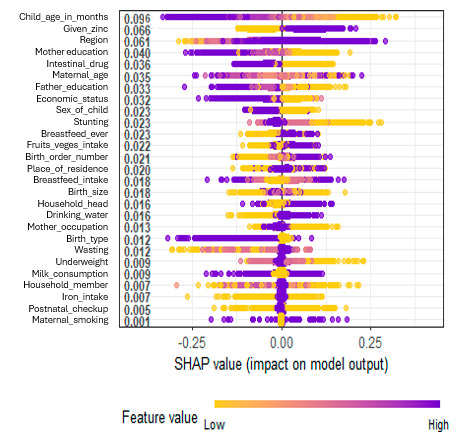
SHAP summary plot on the impact of independent variables on the random forest model’s predictive ability.

Figure 5 illustrates the SHAP (Shapley Additive exPlanations) technique, which provides a global interpretation of each variable’s contribution to the model’s predictive performance. Higher absolute SHAP values indicate a greater influence on the model’s output. The *y*-axis presents variables related to societal, parental, and child characteristics. At the same time, the *x*-axis displays the corresponding SHAP values, highlighting the impact of each feature on the model’s predictions. Feature values are color-coded: yellow represents lower values, and purple represents higher ones. The most influential feature is child age, with a SHAP value of 0.096 indicating a strong positive effect on the model’s forecast. Intake of zinc also shows a high impact on the model output. The region also shows significant effects, suggesting that geographic factors play a role. Parental education—both maternal and father—shows a moderate impact (0.040 and 0.033), underscoring the role of educational attainment in improving child health outcomes. Economic status and maternal age demonstrate slightly lower influence (0.032 and 0.035), suggesting that both maternal factors and household economic conditions contribute to predictive accuracy. Additional significant demographic variables include birth order, breastfeeding history, and stunting, each of which may affect child health outcomes. In contrast, variables such as postnatal checkups and maternal smoking exhibit minimal influence, implying limited predictive utility. Overall, features with SHAP values close to zero have negligible effects, whereas those with values farther from zero exert a more substantial positive or negative impact on the model’s predictions.

**Figure 6:**
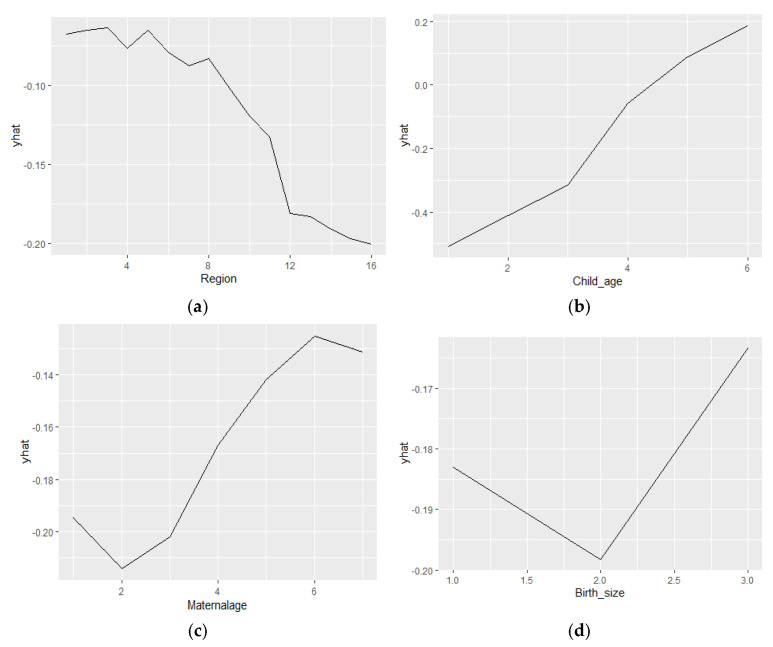

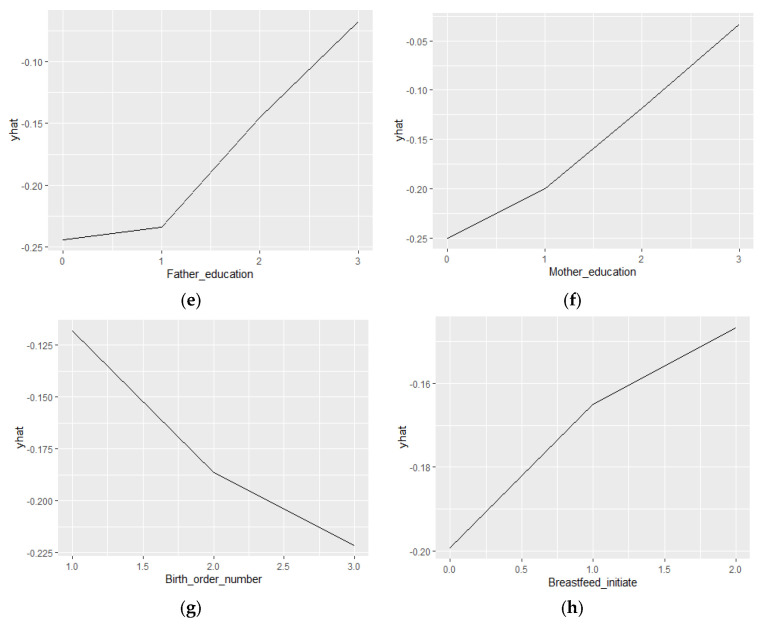
Partial dependence plots of the random forest model’s top features (**a**) Partial dependence plot of the region (**b**) partial dependence plot of child age in months (**c**) partial dependence plot of maternal age (**d**) partial dependence plot of the size of child at birth (**e**) partial dependence plot of the father’s education (**f**) partial dependence plot of the mother’s education (**g**) partial dependence plot of birth order number and (**h**) partial dependence plot of the initiation of breastfeeding.

Figure 6a–h present the partial dependence plots (PDPs) of the top features identified by importance score in the random forest model. Each subplot illustrates the relationship between a specific feature (*x*-axis) and the predicted outcome (*y*-axis, labeled as “yhat”). Figure 6a shows a general downward trend in the values of yhat. In Figure 6b, a clear positive linear trend is observed, indicating that as child age increases, the predicted value also rises. This suggests that older children are associated with a higher predicted risk of childhood anemia, explaining the increased risk of anemia with advancing age. Figure 6c displays a more complex pattern: the predicted value (yhat) begins at approximately −0.20 when maternal age is 2, declines initially, and then gradually increases, surpassing −0.18 around maternal age 6. This trend reveals an inverse association at lower maternal ages, followed by a positive relationship at higher ages. These nuanced trends could help understand maternal health and infant development. Figure 6d demonstrates a two-phase relationship between birth size and predicted childhood anemia. In the first segment (up to a birth size of 1.5), a downward trend is evident, indicating that smaller birth sizes are linked to lower predicted values, which may reflecta higher risk of anemia. Beyond this poin at 2.0t, the trend reverses sharply upward, indicating that larger birth sizes are associated with more favorable health outcomes for anemi, could imply various biological, health and environmental factors at playa. In Figure 6e, a direct, positive association is evident between paternal education and predicted outcomes. Higher levels of fathers’ education correspond to increased predicted values, suggesting a beneficial effect on childhood anemia. A similar pattern is seen in Figure 6f, where improved maternal education levels also show a positive impact on predicted outcomes, further emphasizing the importance of parental education in influencing childhood anemia. Figure 6g reveals a negative relationship between birth order and the predicted outcome. As birth order increases, the predicted value gradually declines, indicating that children born later in birth order may experience comparatively lower health outcomes related to anemia. Finally, Figure 6h shows that, although predicted values remain negative across the range, a positive linear trend is observed as breastfeeding initiation increases. This suggests an inverse relationship between breastfeeding and anemia status—proper breastfeeding initiation corresponds with a lower likelihood of anemia.

## 4. Discussion

Our study is the first of its kind, conducted on a nationwide dataset of 9353 children in Ghana, addressing a specific gap in the application of machine learning techniques for assessing anemia status and essential factors throughout the country. We believe our research contributes to the existing body of knowledge by conducting a multivariate modeling study that incorporates various machine learning techniques, which have not been previously performed in Ghana. In low-resourced countries such as Ghana, where most of the children are malnourished and anemic, accurate assessment of the anemic status of children through machine learning algorithms can play a vital role in reducing the disease burden. The current study uses a quantitative cross-sectional study design based on data from the GDHS survey to examine the prevalence of anemia and the factors associated with it among children of five years and under in Ghana. We performed a binary logistic regression model using suitable machine learning techniques, including random forest, logistic regression, KNN, and decision trees, simultaneously. Findings have shown that more than half (54.20%) of children of five years and under five years experienced anemic disorders.

Among the factors evaluated, paternal education, socioeconomic status, iron intake during pregnancy, and the baby’s postnatal checkup within two months of birth emerged as the significant predictors of a child’s anemia status. Overall, paternal education played a strong protective role in a child’s anemic status, implying that supporting paternal education and improving societal behavior can contribute to improvements in the anemic status of young children. There should be a supportive environment for parents to prioritize their children’s health and adopt healthy feeding practices. In Ghana, there is a need for focused programs to educate parents, raise their awareness about anemia prevention and treatment, and encourage the adoption of healthy behaviors. The government should improve access to healthcare services, particularly in rural or underserved areas.

Our results indicate that children of educated mothers have a lower likelihood of being anemic compared to children of mothers with no formal education. Educated mothers tend to be better aware of health-related information and are better prepared to protect their children against the risk of malnutrition and anemia [39]. Our findings align with numerous previous studies that have shown an educated mother is significantly less likely to have anemic children [25,33,34,35]. However, a significant number of children of mothers with good nutritional knowledge suffered from anemia, demonstrating that although education is essential, other variables also come into play [40]. Nutritional behaviors and food choices are influenced by maternal education. More highly educated mothers often provide their kids with better nutrition, including nutritious food and supplements high in iron, which are essential for preventing iron deficiency anemia [41,42,43]. Our findings show a significant association between a father’s education and early childhood anemia. A child whose father has received primary and secondary education has a lower chance of developing anemia. These findings suggest that higher paternal education may help reduce the risk of iron deficiency in children. Our study found that middle-income and affluent youngsters were less likely to be anemic. This is consistent with other studies showing that families with higher incomes are in a better position to offer nutritious foods and access to medical care, both of which are essential for avoiding anemia [44]. More affluent households may be able to afford a more diverse diet that includes foods high in iron [45,46]. Conversely, low-resource families may rely on less bioavailable iron sources, such as those found in plant-based diets, raising the risk of anemia.

The risk of anemia in children is lower when mothers consume iron during pregnancy. This finding is also supported by a study [47] which reports that such children are less likely to suffer from anemia. The results highlighted that regular supplementation might reduce the chances of low birth weight and maternal anemia, all of which are connected to a higher incidence of childhood anemia. Sunuwar et al. showed that one of the most critical factors in predicting anemia in children aged 6 to 59 months was the mother’s compliance with iron supplementation [26]. Compared to children whose mothers did not take iron supplements, children whose mothers did had a considerably lower risk of being anemic. This implies that to ensure children have sufficient iron throughout their early development, mothers must regularly consume adequate amounts of iron. Previous research has shown that infants born to mothers who maintained adequate hemoglobin levels during pregnancy have better health outcomes, including lower rates of anemia. The evidence supports the notion that maternal nutrition, particularly iron intake, plays a critical role in children’s long-term health [46].

The baby’s postnatal examination, conducted within two months after birth, is another significant contributor to the overall assessment. Our study revealed that early childhood anemia could be reduced if newborns received postnatal care. The American Academy of Pediatrics emphasizes the importance of screening children, particularly those at risk, for anemia. Frequent postnatal examinations enable early anemia identification and timely treatment, both of which are essential for enhancing health outcomes [47].

A random forest algorithm achieves a higher prediction accuracy of 94.74% (95% CI: 90.58–95.84%) compared to other machine learning algorithms, indicating that the child's age, maternal age, father’s education, birth size, mother’s education, and birth order have the highest importance scores. Similarly, the second-best machine learning technique for predicting childhood anemia is logistic regression, with an accuracy of 67.35% (95% CI: 66.20–68.49%). According to Zemariam et al., the best machine learning method for predicting anemia in young Ethiopian girls is the random forest algorithm [48]. Other studies have demonstrated stronger predictive ability of the random forest algorithm than other classifiers, ranging from 82% to 98.4% accuracy [49]. The random forest model has been demonstrated to detect significant predictors, including the mother’s health, household circumstances, and child morbidity [50]. According to other studies conducted in Bangladesh [32] and in Afghanistan [51], the random forest method performs the best when compared to other machine learning algorithms. However, a former study reported that the naïve Bayes model outperforms KNN and support vector machine models with an accuracy of 99% [52]. Anemia is significantly predicted by the child’s age, particularly if the child is between 6 and 23 months old. This age group is more vulnerable to iron deficiency and anemia, according to studies, making it an essential consideration in predictive models [53].

Several factors predicted childhood anemia. Nonetheless, the logistic regression and random forest models differ in their level of significance. The random forest analysis reveals that the following variables are the most significant predictors of anemia in Ghanaian children: region, the child's age in months, maternal age, the father’s education, birth size, the mother’s education, birth order number, and socioeconomic status. In Ghanaian studies on childhood anemia, the region does play a significant role. The Upper East and Upper West regions have consistently had the highest prevalence of anemia among children under five, with rates as high as 88.9% and 88.1%, respectively, in previous surveys. New data continue to identify these regions as high-risk areas [54,55]. Child age in months also has a high importance score. These findings are consistent with various studies. According to research examining data from Ethiopia, the prevalence of anemia was greater in children aged 6–23 months (72.0%) than in older children (50.1%). One known risk factor for childhood anemia is low birth weight. This study emphasized the significance of birth size as a predictor by showing that children born with low birth weight had a greater prevalence of anemia. Maternal age has a crucial role, as children born to mothers under the age of twenty and women over the age of forty have greater incidences of anemia [53] and the research from India showed that children between the ages of 6 and 24 months had a much higher likelihood of being anemic than those between the ages of 36 and 59 months. The children from households with lower levels of education are more likely to suffer from anemia because they have less access to nutrient-dense diets [56]. Although their relative importance fluctuates, both logistic regression and random forest consistently identify the child's age in months and birth order number as significant factors. Nevertheless, the two algorithms found conflicting predictors, such as the region being given considerable value in the RF method and the postnatal checkup being given great importance in logistic regression. Furthermore, maternal smoking was found to be the least significant predictor of childhood anemia in random forest, but the third most significant predictor in logistic regression.

In logistic regression, the postnatal checkup shows a high importance score, indicating that postnatal checkups are essential for newborn children. According to WHO guidelines, receiving thorough care at this time is crucial to avoiding problems [57]. Because of their faster growth and greater need for minerals like iron, younger children, especially those under 24 months, are more likely to develop anemia [58,59]. Zinc has a high importance score; zinc is essential for general health and immunological function. Children’s enhanced hemoglobin levels have been linked to zinc supplementation, illustrating the significance of this nutrient in dietary treatments meant to prevent anemia [60]. Pregnancy-related intestinal health drug consumption may potentially influence childhood anemia. According to research, maternal health interventions, including the use of such drugs, are essential for avoiding anemia in children [8].

### Strengths and Limitations of the Study

A key strength of this study lies in its use of the recent wave of nationally representative data from the Ghana Demographic and Health Survey (GDHS-2022), which incorporates over 27 associated predictors, a broad range of societal, parental, and child-level variables relevant to anemia among children up to five. Previous anemia research conducted in Ghana utilized earlier DHS waves (2014–2016) and employed traditional logistic regression with a limited array of covariates [7,8,54]. In comparison, our research of GDHS-2022 (n = 9353) includes a comprehensive set of predictors at the household, parental, and child levels, evaluates four machine learning methods, and employs SHAP to demonstrate that region remains a significant factor, despite multivariable logistic regression diminishing its importance. After comparing the predictive performance of these models, the study identifies substantial determinants of childhood anemia within the population. This innovative integration of contemporary data and interpretable machine learning differentiates our research.

The performance of each model was assessed using a range of evaluation metrics. Interestingly, while univariate logistic regression identified region as a significant factor associated with anemia, this variable lost significance in the multivariate model. However, the feature importance scores and SHAP (Shapley Additive exPlanations) plots of the best-performing machine learning model, the random forest model, reaffirmed the importance of the region factor. Both of these plots of the machine learning algorithm indicated considerable regional disproportion in anemic prevalence. This finding underscores the need for region-specific intervention strategies.

Moreover, our results suggested that parental characteristics, particularly educational attainment and maternal health behaviors (including iron supplementation during pregnancy), play a more prominent role in predicting anemia than child-related factors. Among child-specific variables, only postnatal checkup within two months emerged as a significant predictor, emphasizing its value for early detection and intervention. These insights contribute to a deeper understanding of the determinants of childhood anemia in Ghana and illustrate the practical value of machine learning approaches for informing targeted public health strategies in similar contexts.

This study has several limitations, which may have influenced the generalizability of the results. First, the secondary data limits our choice of variables in the study. For example, various clinical indicators of anemia, including the pallor of the palms, conjunctiva, and tongue, were absent from the data. Their inclusion could have improved the subpar performance of the machine learning algorithms employed in this study. Studying the use of machine learning algorithms with a richer set of variables to forecast pediatric anemia based on such clinically significant features might be useful in future research. Moreover, information about fever, diarrhea, and cough during the two weeks preceding the study was obtained through interviews with mothers. Such self-reported data, which were not independently verified, could have been prone to several biases, including social desirability bias and recall bias. Another limitation of this study pertains to the missed seed setting, which affects reproducibility. Without setting a seed, there can be inconsistent results, making it challenging to reproduce and exactly compare the two different models. This issue can be addressed in our future work to ensure reproducibility. Regardless of these limitations, the policy and practice-relevant research evidence is valuable due to its strengths. The current study utilizes nationally representative data collected using instruments that have been tested for validity and reliability, ensuring its generalizability and applicability to public health and healthcare interventions. Moreover, this research is innovative in that we employed several machine learning techniques to identify childhood predictors that would have otherwise remained unreported if simple statistical analysis had been applied.

## 5. Conclusions

The high prevalence of childhood anemia in Ghana and its adverse effects on child development make it a significant public health problem. Approximately 49% of Ghanaian children aged 6 to 60 months have anemia, according to current data from the 2022 Ghana Demographic and Health Survey. Given its detrimental impact on cognitive development, physical growth, and general well-being, anemia continues to be a significant public health problem, affecting about half of all children in this age range. The prevalence of moderate anemia in children was approximately 27.6%, and moderate to severe anemia affected about 21.4% of the population. According to this study, factors such as the father’s education, socioeconomic level, the mother’s education, iron consumption during pregnancy, and the baby’s postnatal checkup will have a substantial impact on childhood anemia. It proposes a comprehensive approach to reducing childhood anemia that encompasses social and economic factors, including iron supplementation and postnatal checkups. It also promotes educating pregnant women and their families about health and well-being. The results demonstrated that, in comparison to the other machine learning algorithms applied in this study, the random forest machine learning algorithm provides stronger prediction accuracy, with the logistic regression method ranking second. Additionally, several combinations of the most significant predictors of childhood anemia have been identified using random forest and logistic regression machine learning methods, with distinct levels of significance. Therefore, in addition to the conventional statistical analysis technique, the study provides evidence on how the machine learning approach can be utilized to better identify the determinants of childhood anemia. Healthcare professionals in the primary healthcare unit may use the findings in their day-to-day clinical work at remote primary healthcare units with inadequate diagnostic resources for identifying anemia. By addressing key child, parental, and societal factors influencing anemia, public health programs can develop targeted interventions in different geographical regions to improve the anemia-related health status of children under five. A greater focus should be placed on strategies that involve nutritional awareness and counseling for parents, easy access to healthcare, and the provision of iron supplements to mothers during pregnancy, as well as deworming programs to reduce parasite infections, which can contribute to a reduction in anemia rates.

## Figures and Tables

**Table 1:** Summary statistics of risk factors and univariate analysis of anemia status for children of ages 5 years or below, GDHS-2022, (n = 9353).

Sr. No	Attributes	Categories	freq.	Percentage %	OR (C-I)	*p* Value
**Societal Characteristics**						
1	Region	Western *	454	4.85	-	
		Central	510	5.45	0.002 (−0.001–0.383)	0.991
		Greater Accra	455	4.86	0.369 (0.170–0.530)	0.070
		Volta	383	4.09	0.252 (−0.156–0.661)	0.227
		Eastern	436	4.66	0.309 (−0.212–0.692)	0.132
		Ashanti	592	6.33	0.241 (−0.612–0.329)	0.202
		Western North	434	4.64	0.002 (0.001–0.415)	0.991
		Ahafo	497	5.31	0.398 (−0.788–0.728)	**0.045**
		Bono	427	4.57	0.338 (0.149–0.471)	0.106
		Bono East	659	7.05	0.212 (−0.154–0.579)	0.256
		Oti	632	6.76	0.522 (0.146–0.898)	**0.006**
		Northern	970	10.37	1.996 (1.646–2.345)	**<0.001**
		Savannah	797	8.52	0.639 (0.284–0.995)	**<0.001**
		North East	868	9.28	0.835 (0.4804- 1.190)	**<0.001**
		Upper East	638	6.82	0.770 (0.393–1.1476)	**<0.001**
		Upper West	601	6.43	0.803 (0.432–1.174)	**<0.001**
2	Household members	<4 *	298	3.19	-	
		4–6	117	1.25	1.149 (0.604–2.187)	0.671
		7–9	233	2.49	1.238 (0.731–2.097)	0.425
		>9	8705	93.07	1.041 (0.729–1.487)	0.823
3	Place of residence	Urban *	3857	41.24	-	
		Rural	5496	58.76	1.516 (1.335–1.722)	**<0.001**
4	Source of drinking water	Unimproved *	3862	41.29	-	
		Improved	5491	58.71	1.191 (1.049–1.352)	0.176
5	Sex of household head	Male *	6880	73.56	-	
		Female	2473	26.44	0.831 (0.722–0.956)	**0.010**
6	Socioeconomic status	Poor *	5308	56.75	-	
		Middle	1681	17.97	0.746 (0.629–0.885)	**0.001**
		Rich	2364	25.28	0.449 (0.386–0.522)	**<0.001**
**Parental Characteristics**						
7	Mother’s education	No education *	2917	31.19	-	
		Primary	1496	15.99	0.918 (0.752–1.120)	0.402
		Secondary	4237	45.3	0.529 (0.457–0.614)	**<0.001**
		Higher	703	7.52	0.400 (0.309–0.519)	**<0.001**
8	Father’s education	No education *	2715	33.51	-	
		Primary	868	10.71	0.862 (0.676–1.100)	0.233
		Secondary	3419	42.2	0.543 (0.464–0.636)	**<0.001**
		Higher	1099	13.57	0.485 (0.390–0.603)	**<0.001**
9	Maternal age	15–19 *	353	3.77	-	
		20–24	1671	17.87	0.810 (0.540–1.217)	0.312
		25–29	2276	24.33	0.657 (0.442–0.976)	**0.038**
		30–34	2266	24.23	0.643 (0.433–0.953)	**0.028**
		35–39	1713	18.31	0.541 (0.362–0.809)	**0.003**
		40–44	819	8.76	0.548 (0.358–0.839)	**0.006**
		45–49	255	2.73	0.780 (0.460–1.321)	0.357
10	Maternal smoking	No *	9287	99.29	-	
		Yes	66	0.71	1.293 (0.626–2.671)	0.487
11	Breastfeed ever	No *	4195	44.85	-	
		Yes	5158	55.15	1.754 (1.546–1.991)	**<0.001**
12	Initiation of breastfeeding	Immediately *	3667	63.84	-	
		Within the first hour	1805	31.42	0.926 (0.778–1.104)	0.395
		Within 1 day	272	4.74	0.988 (0.675–1.445)	0.951
13	Mother's occupation	Not working *	1537	16.43	-	
		Working	7816	83.57	0.756 (0.632–0.904)	**0.002**
14	Intake of iron during pregnancy	No *	469	8.99	-	
		Yes	4749	91.01	0.575 (0.410–0.807)	**0.001**
15	Consumption of drugs for intestinal parasites during pregnancy	No *	2382	45.65	-	
		Yes	2836	54.35	0.680 (0.568–0.814)	**<0.001**
**Child Characteristics**						
16	Birth order number	1st born *	2386	25.51	-	
		2–4	4782	51.13	1.164 (0.999–1.355)	0.051
		>5	2185	23.36	1.476 (0.999–1.355)	**<0.001**
17	Birth type	Single birth *	8907	95.23	-	
		Multiple births	446	4.77	0.873 (0.637–1.196)	0.398
18	Sex of child	Male *	4804	51.36	-	
		Female	4549	48.64	0.854 (0.753–0.968)	**0.014**
19	Size of child at birth	Small *	792	13.73	-	
		Average	2329	40.38	0.957 (0.737–1.243)	0.747
		Large	2647	45.89	0.912 (0.705–1.179)	0.484
20	Formula milk consumption	No *	5123	90.5	-	
		Yes	538	9.5	0.771 (0.589–1.009)	0.058
21	Child's age in months	0–6 *	609	13.61	-	
		7–12	490	10.95	1.035 (0.649–1.650)	0.884
		13–24	1017	22.73	1.032 (0.660–1.612)	0.889
		25–36	845	18.89	0.571 (0.365–0.893)	**0.014**
		37–48	834	18.64	0.473 (0.302–0.740)	**0.001**
		49–60	679	15.18	0.342 (0.217–0.539)	**<0.001**
22	Stunting	No *	199	4.45	-	
		Moderate	618	13.83	0.700 (0.481–1.017)	0.062
		Severe	3653	81.72	1.423 (0.301–1.594)	**<0.001**
23	Underweight	No *	107	2.39	-	
		Moderate	477	10.67	0.958 (0.595–1.544)	0.863
		Severe	3886	86.94	0.540 (0.348–0.839)	0.636
24	Wasting	No *	48	1.07	-	
		Moderate	213	4.77	0.873 (0.439–1.736)	0.699
		Severe	4209	94.16	0.777 (0.413–1.460)	0.434
25	Intake of fruits and vegetables	No *	3087	62.29	-	
		Yes	1869	37.71	0.268 (0.055–1.523)	**0.011**
26	Baby postnatal checkup within 2 months	No *	222	5.04	-	
		Yes	4183	94.96	0.572 (0.358–0.915)	**0.020**
27	Given zinc	No *	747	61.89	-	
		Yes	460	38.11	1.219 (0.855–1.739)	0.273

Note: * Indicates the reference category, OR indicates odds ratio, significant *p* values are shown in bold, C-I indicates confidence interval, freq. indicates the frequency, and % indicates the percentage.

**Table 2:** Binary logistic regression analysis of anemia status for children 5 years or below, GDHS-2022, (n = 9353).

Sr No.	Attributes	Categories	AOR (C-I)	*p* Value
**Societal Characteristics**				
1	Region	Western *	-	
		Central	0.363 (−2.044–2.772)	0.767
		Greater Accra	−2.485 (−5.557–0.587)	0.113
		Volta	1.690 (−1.203–4.584)	0.252
		Eastern	−2.033 (−4.890–0.823)	0.163
		Ashanti	−1.042 (−3.341–1.256)	0.374
		Western North	−2.099 (−4.950–0.751)	0.149
		Ahafo	−0.717 (−3.111–1.676)	0.557
		Bono	−1.729 (−4.769–1.309)	0.265
		Bono East	−0.610 (−3.009–1.787)	0.618
		Oti	−0.392 (−2.806–2.021)	0.750
		Northern	0.364 (−1.873–2.601)	0.750
		Savannah	1.446 (−1.176–4.069)	0.280
		North East	0.678 (−1.680–3.037)	0.573
		Upper East	1.996 (−1.180–5.173)	0.218
		Upper West	2.016 (−1.217–5.250)	0.222
2	Household members	<4 *	-	
		4–6	0.143 (0.103–3.420)	0.432
		7–9	0.654 (0.521–2.543)	0.596
		>9	0.342 (0.832–5.321)	0.104
3	Place of residence	Urban *	-	
		Rural	1.791 (0.247–2.951)	0.564
4	Source of drinking water	Unimproved *	-	
		Improved	2.312 (0.272–9.613)	0.442
5	Sex of household head	Male *	-	
		Female	0.457 (0.078–2.672)	0.385
6	Socioeconomic status	Poor *	-	
		Middle	0.010 (0.0001–0.568)	**0.025**
		Rich	0.041 (0.001–1.077)	**0.046**
**Parental Characteristics**				
7	Mother’s education	No education *	-	
		Primary	0.040 (0.0009–1.608)	0.088
		Secondary	0.068 (0.003–1.173)	0.064
		Higher	0.002 (0.0009–0.529)	**0.029**
8	Father’s education	No education *	-	
		Primary	4.945 (0.850–7.058)	0.062
		Secondary	0.652 (0.288–2.126)	**0.009**
		Higher	0.156 (3.582–5.457)	**0.012**
9	Maternal age	15–19 *	-	
		20–24	9.766 (0.396–9.946)	0.163
		25–29	6.270 (0.255–4.070)	0.261
		30–34	2.134 (0.083–5.762)	0.647
		35–39	0.042 (0.0004–4.554)	0.186
		40–44	0.793 (0.009–6.967)	0.919
		45–49	0.486 (0.765–3.210)	0.659
10	Maternal smoking	No *	-	
		Yes	0.672 (0.383–1.895)	0.085
11	Breastfeed ever	No *	-	
		Yes	3.586 (0.228–6.299)	0.363
12	Initiation of breastfeeding	Immediately *	-	
		Within the first hour	3.445 (1.540–7.313)	0.119
		Within 1 day	4.071 (0.104–5.180)	0.065
13	Mother occupation	Not working *	-	
		Working	0.673 (0.056–8.018)	0.755
14	Intake of iron during pregnancy	No *	-	
		Yes	0.017 (0.004–6.122)	**0.033**
15	Consumption of drugs for intestinal parasites during pregnancy	No *	-	
		Yes	0.761 (0.122–4.715)	0.769
**Child Characteristics**				
16	Birth order number	1st born *	-	
		2–4	0.132 (0.009–1.943)	0.140
		>5	0.434 (0.014–3.224)	0.632
17	Birth type	Single birth *	-	
		Multiple births	7.641 (0.009–8.456)	0.552
18	Sex of child	Male *	-	
		Female	0.877 (0.170–4.520)	0.876
19	Size of child at birth	Small *	-	
		Average	2.760 (0.397–4.549)	0.150
		Large	6.603 (0.330–7.788)	0.217
20	Formula milk consumption	No *	-	
		Yes	2.845 (0.073–3.518)	0.575
21	Child age in months	0–6 *	-	
		7–12	0.096 (0.0011–8.606)	0.308
		13–24	0.053 (0.0003–8.935)	0.262
		25–36	1.024 (0.780–1.317)	0.929
		37–48	0.704 (0.472–1.0464)	0.087
		49–60	0.710 (0.435–1.034)	0.074
22	Stunting	No *	-	
		Moderate	3.063 (1.106–4.611)	0.146
		Severe	0.747 (0.851–3.825)	0.758
23	Underweight	No *	-	
		Moderate	1.224 (0.048–3.627)	0.902
		Severe	0.969 (0.995–2.714)	0.840
24	Wasting	No *	-	
		Moderate	0.067 (0.002–1.920)	0.114
		Severe	1.692 (0.383–2.012)	0.555
25	Intake of fruits and Vegetables	No *	-	
		Yes	4.755 (0.639–5.364)	0.128
26	Baby postnatal checkup within 2 months	No *	-	
		Yes	0.732 (3.452–8.076)	**0.010**
27	Given zinc	No *	-	
		Yes	0.505 (0.081–3.130)	0.521

Note: * Indicates the reference category, significant *p* values are shown in bold, C-I indicates confidence interval, and AOR indicates adjusted odds ratio.

**Table 3:** Performance indicators of all four machine learning algorithms, evaluated on training data for the prediction of childhood anemia.

Evaluation Parameters	Random Forest			Decision Tree			Logistic Regression			K-Nearest Neighbor		
Confusion matrix	Predicted			Predicted			Predicted			Predicted		
		**No anemia**	**Anemia**		**No anemia**	**Anemia**		**No anemia**	**Anemia**		**No anemia**	**Anemia**
	**No Anemia**	507	1829	**No anemia**	1716	1169	**No anemia**	1607	1260	**No anemia**	1216	863
	**Anemia**	1888	2330	**Anemia**	1143	2520	**Anemia**	877	2803	**Anemia**	1651	2817
	**% (95% CI)**			**% (95% CI)**			**% (95% CI)**			**% (95% CI)**		
**Accuracy**	94.74 (90.58–95.84)			64.69 (63.51–65.84)			67.35 (66.20–68.49)			61.60 (60.41–62.78)		
**Sensitivity**	82.50 (80.47–86.85)			68.79 (67.27–70.29)			76.16 (74.76–77.54)			63.04 (61.61–64.47)		
**Specificity**	50.78 (48.54–58.92)			59.48 (57.66–61.28)			56.05 (54.21–57.88)			58.48 (56.34–60.62)		
**Positive predictive value**	75.23 (73.65–76.24)			68.31 (66.78–69.810)			68.98 (67.54–70.41)			76.54 (75.15–77.91)		
**Negative predictive value**	56.81 (51.31–58.81)			60.02 (58.2–61.82)			64.69 (62.78–66.58)			42.41 (40.6–44.25)		
**AUC**	86.62 (80.6–88.86)			64.16 (63.61–65.34)			72.47 (71.26–73.7)			59.48 (58.35–60.62)		
**F1 scores**	96.94			57.36			67.82			51.03		
**Performance time**	1.5913 s			1.1774 s			1.1448 s			1.81 s		

**Table 4:** Performance indicators of all four machine learning algorithms, evaluated on test data for the prediction of childhood anemia.

Evaluation Parameters	Random Forest			Decision Tree			Logistic Regression			K-nearest Neighbor		
**Confusion matrix**	**Predicted**			**Predicted**			**Predicted**			**Predicted**		
		**No anemia**	**Anemia**		**No anemia**	**Anemia**		**No anemia**	**Anemia**		**No anemia**	**Anemia**
	**No Anemia**	280	366	**No anemia**	1716	1169	**No anemia**	1607	1260	**No anemia**	1216	863
	**Anemia**	772	1387	**Anemia**	1143	2520	**Anemia**	877	2803	**Anemia**	1651	2817
	**% (95% CI)**			**% (95% CI)**			**% (95% CI)**			**% (95% CI)**		
**Accuracy**	95.75 (99.48–99.89)			64.69 (63.51–65.84)			67.35 (66.20–68.49)			61.60 (60.41–62.78)		
**Sensitivity**	98.74 (99.35–99.93)			68.79 (67.27–70.29)			76.16 (74.76–77.54)			63.04 (61.61–64.47)		
**Specificity**	67.89 (99.29–99.95)			59.48 (57.66–61.28)			56.05 (54.21–57.88)			58.48 (56.34–60.62)		
**Positive predictive value**	88.81 (99.45–99.96)			68.31 (66.78–69.810)			68.98 (67.54–70.41)			76.54 (75.15–77.91)		
**Negative predictive value**	80.67 (99.17–99.91)			60.02 (58.2–61.82)			64.69 (62.78–66.58)			42.41 (40.6–44.25)		
**AUC**	98.34 (99.55–99.93)			64.16 (63.21–65.34)			72.47 (71.26–73.7)			59.48 (58.35–60.62)		
**F1 score**	98.20			59.56			68.27			54.48		
**Performance time**	0.06 s			0.75 s			0.08 s			0.2 s		

## Data Availability

The data set used in the study was taken from the Demographic and Health Survey (DHS) website, and the files are available at the following url: https://dhsprogram.com/data/dataset/Ghana_Standard-DHS_2022.cfm?flag=0 (accessed on 4 July 2025).

## Abbreviations

The following abbreviations are used in this manuscript:

ML	Machine Learning
DT	Decision Tree
KNN	K-nearest Neighbor
RF	Random Forest
AUC	Area Under the Curve
ROC	Receiving Operating Curve
